# How to achieve nutrition goals by actual nutrition guidelines

**DOI:** 10.1186/s13054-019-2502-x

**Published:** 2019-06-13

**Authors:** Christian Stoppe, Jean-Charles Preiser, Daren Heyland

**Affiliations:** 1 Department of Intensive Care Medicine, RWTH Aachen University, University Hospital of the RWTH Aachen, Pauwelsstraße 30, 52074 Aachen, Germany; 2 Department of Intensive Care, Erasme University Hospital, 808 route de Lennik, Brussels, Belgium; 3 Department of Critical Care Medicine, Queen’s University and Clinical Evaluation Research Unit, Angada 4, Kingston General Hospital, Kingston, Ontario K7L 2V7 Canada

While more patients are surviving the hospitalization, ICU survivors frequently experience significant post-ICU morbidities including muscle weakness and impairments in physical functioning that can persist for years and results in significant healthcare-associated costs. One major factor contributing to this “post-ICU disability” is the loss of functional lean body mass, highlighting the importance of adequate nutrition support as an integral component in the treatment of critically ill patients. High protein intakes are expected to stimulate new protein synthesis, thereby preserving muscle mass [1]. Recent randomized trials demonstrated that providing increased total calories *alone* to ICU patients may not improve outcomes [2–4]. However, observational studies report that optimizing daily protein intake, rather than total caloric intake, decreases infections, mechanical ventilation duration, time to discharge, and mortality [5].

Enteral nutrient delivery is often impaired by gastrointestinal intolerance, fasting for diverse medical procedures, and lack of feeding protocols which belong to the major reasons why less than 60% of recommended protein intake is usually delivered to the general ICU patients [6]. Historically, the feeding protocol has largely been based on an hourly “rate-based” feeding (RBF) approach, while strategies about how to compensate these commonly occurring interruptions are lacking. Consequently, almost a decade ago, Heyland and colleagues introduced a novel enteral feeding protocol designed to overcome the main barriers to adequate delivery of enteral nutrition, the Enhanced Protein-Energy Provision via the Enteral Route Feeding Protocol (PEPuP protocol) [7]. The main component of this innovative protocol was a switch from RBF to volume-based feeds where the nutritional targets were expressed in a volume per day of a nutritional solution needed to achieve the protein per energy targets. The PEPuP protocol results in 12–15% increase in the amount of protein and calories received by the patient in the context of a cluster randomized multicenter trial [8].

With this background, Brierly-Hobson et al. have conducted a before-after study of implementing VBF in their “real-world” setting. They demonstrated that a comprehensive training of dieticians and immediate initiation of this feeding strategy represent key factors for success and that the implementation of this nutrition strategy is feasible and effectively increases the caloric and protein intake of critically ill patients. The magnitude of the nutritional improvements seen here is with 20% more protein delivered in the VBF-based group which is significantly higher than the rate-based group and comparably effective as the so-called PEPuP protocol [8]. Yet, although a significant increase of protein intake was achieved by using this protocol, not all their patients received optimal protein intake (> 80%) of the aimed target, which pose the question why the investigators did not consider the use of protein supplements. In a recent study, O’Keefe et al. demonstrated that the combined use of empiric EN protein supplement is safe, when used in combination with EN in critically ill patients, and reaches 2 g/kg/day of protein intake per day [9]. In fact, enteral protein supplementation is one of a number of possible ways which has previously been demonstrated to increase protein intake in critically ill patients [8]. Alternatively, the combined use of enteral and parenteral nutrition has previously been demonstrated to significantly increase the protein intake, whereas its clinical relevance still remains unknown [10]. However, the optimal timing of increasing protein intakes is still a matter of debate [1]. The provision of high protein intakes during the early phase of critical illness has been associated with detrimental effects [11], possibly related to an increased production of glucagon and oxidation of amino acids [12], or inhibition of autophagy [10]. Arguably, these latter findings were reported from cohorts of patients at low nutritional risk patients.

Indeed, nutrition support is thought to be of special relevance and as such recommended in patients with high severity of illness, with nutritional high risk, and with prolonged ICU stay [13]. In contrast to these findings, several RCTs demonstrated the safety of high-dose protein application [14] even in the early phase of acute critical illness [15].

One thing that everyone can agree on is that we need more RCTs in nutritionally high-risk patients to be sure of the optimal protein dose in this context. The low level of evidence argues for a formal comparison of the risk-to-benefit ratios of different amounts of protein intakes. The registry-based EFFORT trial is an example of such a study (NCT03160547). In this trial, nutritionally high-risk patients are randomized to usual protein dose (≤ 1.2 g/kg/day) or a higher protein dose (≥ 2.2 g/kg/day). In order to achieve the desired level of protein intakes, the systematic use of volume-based feeding protocols should be advocated as the standard of care, instead of RBF, in both groups, to increase the changes that patients in both groups achieve at least 80% of what has been prescribed. Figure 1 illustrates VBF as strategies to improve enteral nutrition delivery, when compared to RBF. Then, with additional enteral protein supplements or parenteral nutrition or intravenous amino acids, patients in the high-dose group will be able to reach the higher dose targets. We eagerly await the results of such informative trials to provide more information on the clinical impact of such a feeding strategy. In the meantime, to prevent ongoing under-delivery of protein, we recommend that VBF becomes the standard of care in clinical practice. Tools to assist in the implementation can be found on www.criticalcarenutrition.com.

**Fig. 1:**
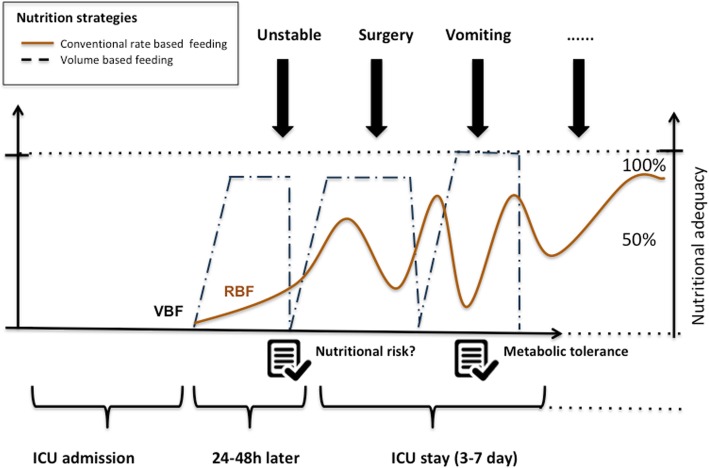
Volume-based feeding as strategies to improve enteral nutrition delivery. ICU intensive care unit, VBF volume-based feeding, RBF rate-based feeding

## Data Availability

Not applicable
